# Historical Clinical Outcomes of Children with Myelomeningocele Meeting the Criteria for Fetal Surgery: A Retrospective Cohort Survey of Brazilian Patients

**DOI:** 10.1055/s-0042-1742404

**Published:** 2022-02-09

**Authors:** Fernando Maia Peixoto-Filho, Tatiana Protzenko Cervante, Antonio Rosa Bellas, Saint Clair Gomes Junior, Denise Araújo Lapa, Gregório Lorenzo Acácio, Paulo Roberto Nassar de Carvalho, Renato Augusto Moreira de Sá

**Affiliations:** 1 Instituto Fernandes Figueira, Rio de Janeiro, RJ, Brazil.; 2 Department of Fetal Medicine, Hospital Israelita Albert Einstein, São Paulo, SP, Brazil.; 3 Department of Obstetrics and Gynecology, Universidade de Taubaté, São Paulo, Brazil.

**Keywords:** meningomyelocele, fetal surgery, myelomeningocele, cerebrospinal fluid shunts, spinal dysraphism, meningomielocele, cirurgia fetal, mielomeningocele, derivações do líquido cefalorraquidiano, disrafismo espinhal

## Abstract

**Objective** To analyze the historical clinical outcomes of children with myelomeningocele (MMC) meeting the criteria for fetal surgery, but who underwent postnatal primary repair.

**Methods** Data from children undergoing postnatal MMC repair between January 1995 and January 2015 were collected from the Neurosurgery Outpatient Clinic's medical records. Children were included if they had ≥ 1 year of postoperative follow-up and met the criteria for fetal surgery. The children's data were then stratified according to whether they received a shunt or not. The primary outcome was mortality, and secondary outcomes were educational delays, hospitalization, recurrent urinary tract infections (UTIs), and renal failure.

**Results** Over the 20-year period, 231 children with MMC were followed up. Based on clinical data recorded at the time of birth, 165 (71.4%) qualify of fetal surgery. Of the 165 patients, 136 (82.4%) underwent shunt placement. The mortality rate was 5.1% in the group with shunt and 0% in the group without, relative risk (RR) 3.28 (95% confidence interval, 95% CI, 0.19–55.9). The statistically significant RRs for adverse outcomes in the shunted group were 1.86 (95% CI, 1.01–3.44) for UTI, 30 (95% CI, 1.01–537) for renal failure, and 1.77 (95% CI, 1.09–2.87) for hospitalizations.

**Conclusion** Children with MMC qualifying for fetal surgery who underwent shunt placement were more likely to have recurrent UTIs, develop renal failure, and be hospitalized. Since approximately half of the shunt procedures could be avoided by fetal surgery, there is a clinical benefit and a possible financial benefit to the implementation of this technology in our setting.

## Resumo

**Objetivo** Analisar os resultados clínicos históricos de crianças com mielomeningocele (MMC) com critérios para cirurgia fetal, mas que foram submetidas a cirurgia pós-natal.

**Métodos** Dados de crianças submetidas à correção de MMC pós-natal entre janeiro de 1995 e janeiro de 2015 foram coletados nos prontuários do Ambulatório de Neurocirurgia. Foram incluídas crianças se tivessem ≥ 1 ano de acompanhamento pós-operatório e atendessem os critérios para cirurgia fetal. As informações dessas crianças foram então estratificadas de acordo com se receberam ou não derivação do líquido cefalorraquidiano. O desfecho primário foi a mortalidade e os desfechos secundários foram atrasos educacionais, hospitalização, infecções recorrentes do trato urinário e insuficiência renal.

**Resultados** Durante o período de 20 anos, 231 crianças com MMC foram acompanhadas. Com base nos dados clínicos registrados no momento do nascimento, 165 (71,4%) atendiam critérios para a cirurgia fetal. Dos 165 pacientes, 136 (82,4%) foram submetidos à colocação de derivação do líquido cefalorraquidiano. A taxa de mortalidade foi de 5,1% no grupo com derivação do líquido cefalorraquidiano e 0% no grupo sem risco relativo (RR) 3,28 (intervalo de confiança 95%, IC 95%, 0,19–55,9). Os RRs estatisticamente significativos para resultados adversos no grupo com derivação do líquido cefalorraquidiano foram 1,86 (IC 95%, 1,01–3,44) para infecção do trato urinário, 30 (IC 95%, 1,01–537) para insuficiência renal e 1,77 (IC 95%, 1,09–2,87) para hospitalizações.

**Conclusão** Crianças com MMC com critérios para cirurgia fetal submetidas à colocação de derivação do líquido cefalorraquidiano eram mais propensas a ter infecções recorrentes do trato urinário, desenvolver insuficiência renal e serem hospitalizadas. Como aproximadamente metade dos procedimentos de derivação poderiam ser evitados por cirurgia fetal, há um benefício clínico e um possível benefício financeiro com a implementação dessa tecnologia em nosso meio.

## Introduction

The worldwide incidence of neural tube defects (NTDs) ranges from 1 to 2 in every 1,000 live births. 1 In Brazil, the prevalence of NTD is estimated between 1.4 and 1.5 in 10,000 births. 2 Myelomeningocele (MMC) is the most common NTD, and is characterized by a dorsal midline lesion composed of a neural plaque (placode) adherent to the adjacent dysplastic epithelial tissue. 2 In the early 1950s, the survival rate of individuals with MMC was below 10%. 3 Currently, one-year survival approaches 90%, and 75% for reaching adulthood. 1

Before 2011, surgical repair of the spinal defect after birth was the only alternative for MMC treatment, with unfavorable results. Clinical evidence in animal models suggests that antenatal MMC repair may favor neurological development. However, fetal surgery increases the risks to maternal health, which do not exist in neonatal surgery. 3 After the Management of Myelomeningocele Study (MOMS trial), a prospective randomized clinical trial comparing antenatal and postnatal repair, antenatal surgery became universally accepted. 4

Prenatal surgery also resulted in improvement of the composite score for mental development and motor function at 30 months ( *p* = 0.007) as well as improvement in several secondary outcomes, including hindbrain herniation by 12 months and ambulation by 30 months. 4 The MOMS trial was the main study demonstrating the results of correction of open NTDs during the prenatal period; the rates of shunt placement were 40% in the prenatal surgery group and 82% in the postnatal surgery group—relative risk (RR), 0.48; 97.7% CI, 0.36 to 0.64; *p* < 0.001. 4 Currently, antenatal surgery for MMC is the standard of care in both the USA and Brazil.

Neurological damage in MMC appears to be primarily a consequence of the development of a defect in the spinal cord, followed by secondary events from exposure of the nervous tissue to the intrauterine environment, known as the two-hit hypothesis. MMC may be associated with other conditions, such as abnormalities of the rhombencephalon, hydrocephalus, and Arnold-Chiari type II malformation, and can have severe consequences, such as motor and sphincter disorders and orthopedic deformities. 5 In 25% of the cases, hydrocephalus related to MMC was present at birth; it can also develop after surgical correction. 6 The standard treatment for hydrocephalus consists of shunt placement, which is required in approximately 80% of the cases. Complications related to shunts contribute to the high morbidity and mortality associated with MMC. In some case series, 52 to 64% of MMC patients undergoing shunt placement experience shunt system failures. 6 7 At 6 years of follow-up, 20% of patients required multiple revisions of their shunts; at 20–25 years of follow-up, 95% had undergone ≥1 revision. 7

Considering that most morbidity and mortality in children with MMC is a consequence of shunt complications, and that fetal surgery can avoid the need for shunt placement in up to half of MMC cases, we aimed to investigate the historical clinical outcomes of children with MMC who meet all current eligibility criteria for fetal surgery, but who were treated by conventional postnatal surgery at our center for a period of over 20 years (1995–2015).

## Methods

**Fig. 1:**
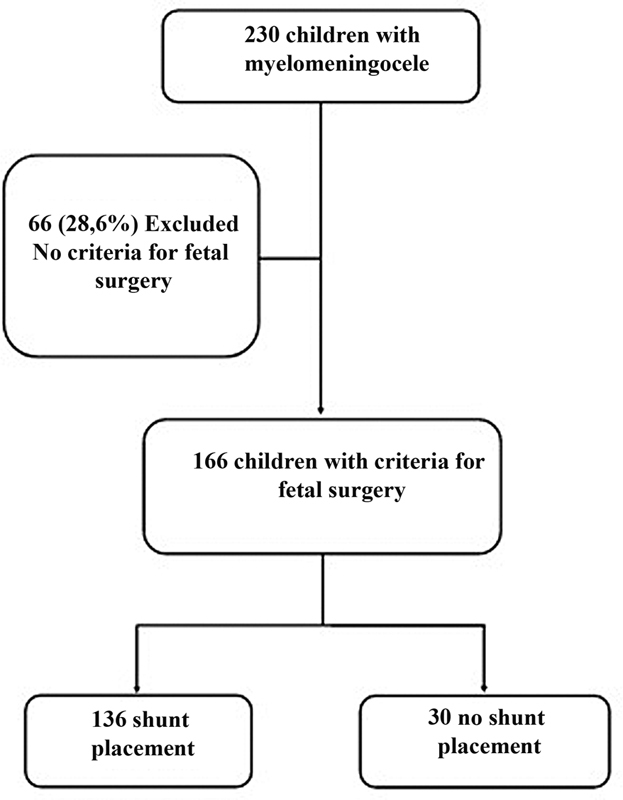
Flowchart of the patients in the study.

This was a cohort study conducted to analyze fetuses that underwent postnatal MMC repair, had ≥1 year of postoperative follow-up, and met the inclusion criteria for the MOMS fetal surgery clinical trial. 4 The newborns included in the study were required to have MMC anatomical levels between T1 and S1. Newborns with congenital infections, other congenital malformations, abnormal karyotypes, multiple gestations, and kyphosis > 30 degrees were excluded from the cohort. 4 The data of the remaining children were then stratified according to whether they received a shunt or not ( Fig. 1 ).

Variables related to newborn gestational age, gender, head circumference (HC), birth weight (BW), MMC anatomical and functional levels, and clinical outcomes were retrieved from medical records and imaging studies. The anatomical level of the MMC of each patient was classified according to radiographic findings from notations in the medical records: thoracic (above T12), thoracolumbar, upper lumbar (L1-L2), lower lumbar (L3-L5), and sacral. The functional levels of MMC were categorized according to the Hoffer et al. scale, as follows: thoracic when lower limb movement was not observed; upper lumbar when psoas, adductor, and/or quadriceps muscle function was identified; lower lumbar when activity of adductors, psoas, quadriceps, medial knee flexors, and, possibly, the anterior tibial and gluteus medius muscles were present; and sacral when the function of all previously mentioned muscles, as well as function of plantar flexors and hip extensors, was present. Clinical outcomes, including recurrent urinary tract infections (UTIs), number of hospitalizations, and date and cause of death were obtained from the medical records. 8

The clinical outcomes studied were mortality, recurrent UTI, asymptomatic bacteriuria, renal failure, fecal incontinence, and hospitalizations.

Descriptive analyses were performed to obtain the frequencies and measures of central tendency (mean and median) of the analyzed variables. Bivariate analysis was used to measure the association among the exposure factors of assessed clinical outcomes (mortality, recurrent UTI, asymptomatic bacteriuria, renal failure, fecal incontinence, and hospitalizations). For numerical variables, the Student *t* -test—for normal distributions—or the Mann-Whitney test were performed. Categorical variables were assessed using chi-square or Fisher's test. For all analyses, a significance level of 0.05 and a confidence interval of 95% were considered. The database was generated using Epi-Info (CDC. Atlanta, Georgia, USA), and statistical calculations were performed using the Statistical Package Social Sciences for Windows (IBM Corp., Armonk, NY, USA) software, version 13.0.

The study was approved by the Local Ethics Committee (CAAE: 52459515.7.0000.5269).

## Results

**Fig. 2:**
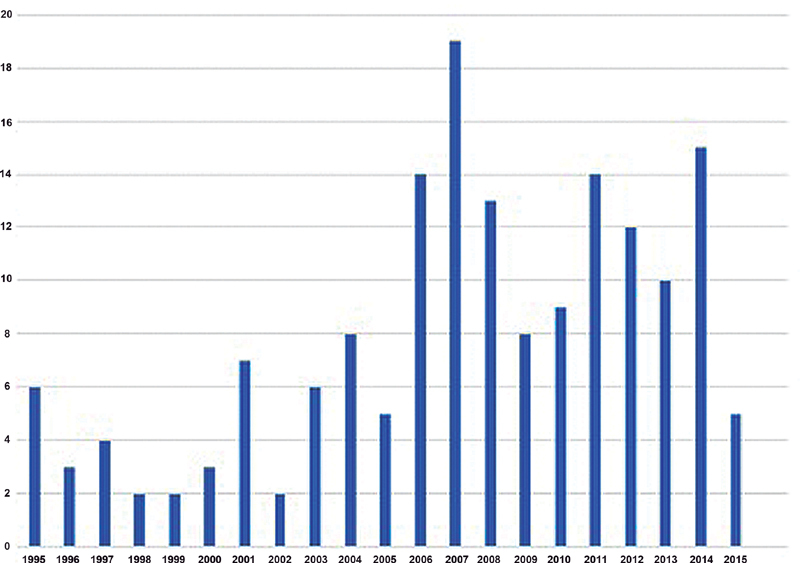
Time distribution of children with myelomeningocele that would now meet criteria for fetal surgery.

For the 20-year period covering births from January 1995 to January 2015, we reviewed the cases of 231 children with MMC undergoing neurosurgery. Of these, 165 (71.4%) met all current eligibility criteria of qualification for fetal surgery ( Fig. 1 ). One hundred and fifty children (90.9%) presented with hydrocephalus, and 156 (94.5%) with Arnold-Chiari malformation. Upper lumbar MMC was present in 29 children (17.6%), lower lumbar MMC in 115 children (69.7%), and sacral MMC in 21 children (12.7%). Functional level analysis revealed that most children, 61 (36.9%), presented a lower lumbar motor level, followed by an upper lumbar motor level in 50 (30.3%), thoracic motor level in 36 (21.8%), and sacral motor level in 13 (7.8%) ( Fig. 2 ).

The average gestational age in the group receiving a shunt was 38.1 weeks (standard deviation, SD, 1.4 weeks), and the mean weight was 3,068 g (SD, 529 g). Comparing the group of children who received a shunt with the group that did not, there was no statistically significant difference in the gestational age ( *p* = 0.40) or mean birth weight ( *p* = 0.992). Among those who received a shunt, females were most prevalent (52.6%), while in the group without shunt, we observed more male children (51.7%), although the difference was not statistically significant ( Table 1 ). In the group undergoing ventricular shunt placement (136–82.4%), 18.4% had defects at the upper lumbar level, 72.1% at the lower lumbar level, and 9.6% at the sacral level. In the group that did not undergo shunt placement, the defect was at the upper lumbar level in 13.8% of the cases, at the lower lumbar level in 58.6%, and at the sacral level in 27.6% of the cases. Comparing the two groups according to the MMC's anatomical level, the differences were statistically significant ( Table 1 ).

**Table 1:** Characteristics of fetuses who met eligibility criteria for fetal surgery, depending on ventricular shunt placement

Variables	No shunt			N	Received a shunt		*p* -value
	*N*	Mean	SD		Mean	SD	
Gestational age (in weeks)	29	37.8	1.16	136	38.1	1.4	0.40
Weight at birth (in grams)	29	3,070	640	136	3,068	529	0.982
	***N***	**%**		**N**	**%**	**95% CI**	***p*** **-value**
Gender							
Female	14	48.3		72	52.6	(5.33–2.65)	0.688
Male	15	51.7		64	47.4	(0.44–1.68)	0.414
Anatomic level of the MMC							
Upper lumbar	4	13.8		25	18.4		0.30
Lower lumbar	17	58.6		98	72.1		0.53
Sacral	8	27.6		13	9.6		0.44
Functional level of the MMC							
Thoracic	1	3.4		35	26.7	(7.98–31.9)	0.000
Upper lumbar	6	20.7		44	33.6	(-5.5–27.1)	0.15
Lower lumbar	18	62.1		43	32.8	(9.36–46.2)	0.003
Sacral	4	13.8		9	6.9	(-3.1–23.9)	0.22
Hydrocephalus	14	48.2		136	100	(0.05–0.15)	0.000
Arnold-Chiari malformation	22	75.9		134	98.5	(4.1–109)	0.000

Abbreviations: 95% CI, confidence interval; MMC, myelomeningocele; SD, standard deviation.

With respect to the functional level of the MMC, thoracic functional level defects were significantly more prevalent among those who received shunts (26.7%) than among those who did not (3.4%) ( *p* = 0.001), while lower lumbar functional level defects were significantly more prevalent among those who did not receive shunts (62.1% versus 32.8%) ( *p* = 0.003). At the upper lumbar and sacral functional levels, the differences were not statistically significant. The prevalence of an upper lumbar functional level was of 33.6% among those receiving shunts, and 20.7% among those who did not undergo shunt placement ( *p* = 0.15). Among those with sacral defects, 6.9% received shunts, and 13.8% did not. The primary outcome (mortality rate) was 5.1% in the group with shunt and 0% in the group without (RR: 3,28 (95% CI, 0.19–55.9). We observed a higher frequency of secondary outcomes (mortality, recurrent UTI, asymptomatic bacteriuria, renal failure, fecal incontinence, and hospitalizations) in the group that underwent shunt placement. However, not all of these differences were statistically significant. The statistically significant RRs for adverse outcomes in the shunted group were as follows: RR, 1.86 (95% CI, 1.01–3.44) for UTI; RR, 30 (CI, 1.01–537) for renal failure; and RR, 1.77 (CI, 1.09–2.87) for hospitalizations ( Table 2 ).

**Table 2:** Clinical outcomes in fetuses that met eligibility criteria for fetal surgery, depending on ventricular shunt placement

Variables	No Shunt		*N*	Received a shunt		RR
	*N*	%		%	95% CI	
Recurrent UTIs	8	27.6	69	51.5	(1.01–3.44)	1.86*
Asymptomatic bacteriuria	91	67.4	17	58.6	(0.82–1.59)	1.14
Renal failure	0	0	2	1.5	(1.6–537.1)	30.0*
Fecal incontinence	19	65.5	101	76.5	(0.88–1.54)	1.16
Educational delay	7	25	60	45.1	(0.92–3.52)	1.80
Hospitalizations	11	37.9	91	67.4	(1.09–2.87)	1.77*
Mortality	0	0	7	5.1	(0.19–55.9)	3.28

Abbreviations: 95% CI, 95% confidence interval; RR, relative risk; UTI, urinary tract infection. Note: *statistically significant.

## Discussion

The purpose of this study was to investigate the clinical outcomes of children with MMC who met all current eligibility criteria for fetal surgery, but who were treated by conventional postnatal surgery with shunt placement. In our cohort, the data clearly demonstrated that children who received a shunt had poorer outcomes. In this group, we observed higher rates of all studied adverse clinical outcomes, except for asymptomatic bacteriuria. However, only the increased risk of recurrent UTIs (RR, 1.86), renal failure (RR, 30), and hospitalizations (RR, 1.77) were statistically significant. The treatment of MMC classically consists of postnatal spinal canal closure surgery and life-long support, as MMC is directly associated with significant motor and cognitive impairment. 3 The MOMS trial was the most important study demonstrating that correction of open NTDs during prenatal care decreases the risk of hydrocephalus and improves motor outcomes at 30 months of age. 5 The prospect of intrauterine treatment being able to improve the long-term clinical outcomes of children with MMC has led to pilot fetal surgery trials in several countries, including Canada, France, and Italy. 9 10 11 In the context of these international trials, our retrospective descriptive survey of 20 years of MMC care provides data on past treatments in children, who did and did not receive shunts, and their clinical outcomes, which will help us to understand the potential benefits of fetal surgery in Brazil ( Table 3 ).

**Table 3:** Comparison of outcomes: fetoscopic, open fetal, and study data from postnatal myelomeningocele repair compared to whether they received a shunt

Type of myelomeningocele repair	Shunt placement
Fetoscopic	44%
Open	34%
Postnatal – present study data	82%

**Source:** Adapted from Kabagambe et al. 19

One of the challenges in comparing our data with that of the other countries and extrapolate its benefits is that abortion is illegal in Brazil, and MMC is not one of the sanctioned indications for medical interruption of a pregnancy.

In our cohort, we found that 71% of patients were candidates for fetal surgery. This relatively high percentage contrasts with the Canadian cohort of 158 MMC cases, where 83 (53%) candidates were elected to terminate the pregnancy and 46 (29%) were deemed candidates for fetal surgery. 9 Thus, differences in access to legally sanctioned pregnancy interruption may confound comparisons across countries. As intrauterine surgery becomes more efficacious and more widely available, fewer women may elect to interrupt their pregnancies.

Our understanding of the protection afforded by spinal cord coverage also comes from the analysis of some less severe variants of spinal dysraphisms, and has led to research on intrauterine correction. 12 Primary failure during the embryonic period—whether closure of the neural tube or of the caudal neuropore—results in exposure of the developing spinal cord to the uterine environment. The absence of the protection afforded by the tissue covering the spinal canal during gestation can lead to the secondary destruction of the exposed nervous tissue, which may occur due to trauma or direct contact with the amniotic fluid. 13

Unfortunately, there are maternal and fetal risks associated with the hysterotomy required for this procedure, which requires developing less invasive methods. 14 For instance, percutaneous fetoscopic techniques to correct open NTDs are under development, 15 16 17 18 although the data demonstrating a favorable risk–benefit relationship for the fetoscopic approach, when compared with the open uterus technique, are still limited. Moreover, the fetoscopic technique continues to be associated with higher rates of prematurity. ( Table 3 ). 19

The screening process for MMC prenatal closure occurs before 27 weeks, and many of our cases could be lost and would not benefit from fetal intervention. Another important challenge is to identify by ultrasound the level of the lesion, 20 which reinforces the need for intensive training program for ultrasound evaluation of these cases. Precise identification of MMC level is very important for preoperative assessment, since fetal repair is offered only to cases in which the upper boundary is located between T1 and S1. The findings of our study allow us to estimate the need for a local fetal surgery program for MMC. Estimates from other countries cannot be readily extrapolated to Brazil, and in addition to adjusting for local incidence, our calculations need to consider the impossibility of interrupting MMC pregnancies. Our data, together with those by Werner et al. 21 in 2012, which showed that fetal surgery in the United States saves US$ 2,066,778 for every 100 cases operated in the prenatal period, supports the implementation of a fetal surgery program for MMC in Brazil.

## Conclusion

Children with MMC qualifying for fetal surgery but who receive shunts are more likely to be hospitalized, have recurrent UTIs, and develop renal failure. Since approximately half of the shunt procedures could be avoided by fetal surgery, there is a clinical benefit and a possible financial benefit to the implementation of this technology in our setting.
